# Organic waste recycling application increases N availability and mitigates N_2_O emission without crop yield penalty in the North China Plain

**DOI:** 10.3389/fpls.2024.1446277

**Published:** 2024-09-17

**Authors:** Lin Chen, Hailun Du, Qing Liu, Wangsheng Gao, Jixiao Cui, Yuanquan Chen

**Affiliations:** ^1^ College of Agronomy and Biotechnology, China Agricultural University, Beijing, China; ^2^ Heilongjiang Academy of Black Soil Conservation and Utilization, Heilongjiang Academy of Agricultural Sciences, Harbin, China; ^3^ National Engineering Research Center of Wheat and Maize, Shandong Academy of Agricultural Sciences, Jinan, China; ^4^ Institute of Environment and Sustainable Development in Agriculture, Chinese Academy of Agricultural Sciences, Beijing, China

**Keywords:** straw, pig manure, biogas residue, nitrogen utilization, N_2_O emission

## Abstract

**Introduction:**

Agricultural organic waste recycling can supply nutrients for crop production and partially replace chemical nitrogen fertilizers, which is beneficial for waste management and environmental protection. Nevertheless, comprehensive evaluation of the effects of different organic materials applications on crop yield and the environment is limited.

**Methods:**

Therefore, in this study, a comprehensive investigation of the synergistic effects of straw, pig manure, and biogas residue recycling on the wheat (*Triticum aestivum L.*) and maize (*Zea mays L.*) systems was carried out in the North China Plain. Field experiments were conducted from 2019 to 2021, comprising five treatments: straw (ST), pig manure (PM), and biogas residue (BR) partially replacing chemical nitrogen fertilizer, sole application of chemical nitrogen fertilizer (CF), and a control with no nitrogen application (WN).

**Results and discussion:**

The results showed that organic materials significantly increased soil total nitrogen (3.04%–9.10%) and N recovery efficiency (RE_N_; 42.21%–44.99%), but pig manure was more beneficial in increasing crop yields (3.50%), especially wheat yields (8.72%), and RE_N_ was significantly higher than that of the other treatments. Organic materials performed differently in wheat and maize seasons, and wheat yield could be improved by organic materials return. Organic materials stimulated N_2_O emission in wheat season (4.28%–32.20%), while biogas residue inhibited the N_2_O emission in maize season (47.47%). The negative effect of straw and biogas residue on yield decreased with increasing years of return, and pig manure continued to contribute to yield. In conclusion, pig manure is the optimal alternative that can increase crop yield, soil N content, and RE_N_ without stimulating N_2_O emissions.

## 1 Introduction

Agricultural production not only provides basic protection for humanity but also produces a large amount of organic materials waste (Wang et al., 2020). The situation is more severe nowadays due to the rapid population and economic growth, which has led to an increase in agricultural waste production capacity (Wang et al., 2018). Moreover, to meet the demands of a growing population, livestock and crop production has increased dramatically, which has further led to the generation of agricultural waste (Liu et al., 2019b). However, most of them are not effectively disposed of or utilized (Zhao et al., 2020a), which has caused a series of environmental pollution problems and has become a thorny global ecological and environmental issue (Alan and Köker, 2023).

The resource utilization of these agricultural wastes is an important means to solve the problem, especially for China, one of the countries with the largest agricultural production and population. It is estimated that the annual output of straw waste is as high as 900 million tons, of which about 30% is not utilized (Chang et al., 2021). The annual output of livestock and poultry manure is up to 3.8 billion tons, with a comprehensive utilization rate of less than 60% (Baldi et al., 2021). Besides the common wastes such as crop residues and animal manure, diversification of agricultural production in China also produced a variety of other organic wastes such as mushroom residue, biogas residue, and wine residue. These different types of agricultural waste are not properly used, resulting in a series of environmental pollution problems as well. Meanwhile, these agricultural organic wastes are rich in organic matter and a variety of nutrients such as nitrogen, phosphorus, and potassium (Meng et al., 2018). Therefore, agricultural waste, when used as agricultural organic material, will play an important role not only in improving the quality of agricultural products but also in improving soil quality and replacing chemical fertilizers. However, farmland production and breeding are separate in China, that is, farmland production relies heavily on fossil energy inputs, while a large amount of waste generated by animal husbandry is not recycled. The heavy use of chemical fertilizers, pesticides, and other non-organic substances, as well as the waste of agricultural organic materials not only exacerbates the fossil energy and other nonrenewable resource inputs but also exacerbates the environmental pollution and greenhouse gas emissions (Wang et al., 2022). Therefore, through the development of recycling agriculture, a large amount of agricultural waste resources can be “turned into treasure”, which is an important path to cope with energy saving and emission reduction in agriculture (Gao, 2007).

There are a lot of benefits to returning organic materials to the field. The application of organic materials, such as crop residues and manure, has been noted to improve soil fertility (Yuan et al., 2023), increase soil organic matter content (Oldfield et al., 2018), and even suppress N_2_O emissions under some conditions (Zhang et al., 2020). Simultaneously, returning organic materials such as straw (Ram et al., 2013) and poultry manure (Bhattacharyya et al., 2007; Li et al., 2021) to soil can increase crop yield. A meta-analysis showed that returning straw to the field increased the average yield of rice, wheat, and maize yields by 5.04%, 8.09%, and 8.71%, respectively (Liu et al., 2023). Moreover, returning agricultural wastes can partly replace the application of chemical fertilizer (Chen et al., 2020; Meng et al., 2021). China’s agricultural production has been facing challenges from the conflicts between population explosion and arable land limitation (Bryan et al., 2018). To mitigate the yield gaps, excessive chemical nitrogen fertilizer was applied in China (Chen et al., 2014a), with the consumption of nitrogen fertilizer up to 17.45 million tons in 2021 (NBSC (National Bureau of Statistics of China), 2022). The extensive application of chemical nitrogen fertilizers has caused environmental concerns such as greenhouse gas emissions and water eutrophication (Galloway et al., 2008). Some studies have reported that organic material returned to the field replaces 70% of chemical fertilizers and results in higher yields than inorganic fertilizers alone (Qaswar et al., 2022) because the mixing of manure with chemical fertilizers not only provides essential nutrients but also extends the duration of fertilizer efficiency (Qaswar et al., 2022). Furthermore, the nutrients in organic materials are released more slowly than chemical fertilizers, which potentially increase the nitrogen recovery rate of the crop and allow for a fuller use of the applied fertilizer (Yang et al., 2015). However, research on organic matter substitution and nutrient utilization is currently inadequate.

Returning organic materials to the field also brings some worries, of which the most current concern is whether it causes greenhouse gas, mainly N_2_O, emissions. Several studies have discussed the influences of different organic matters on N_2_O emissions, but there are inconsistencies in their results. For example, Wang et al. (2019) observed that straw return increased N_2_O emissions in the rice–wheat system, while Liu et al. (2019b) showed that straw incorporation suppressed N_2_O emissions in the wheat growing season. The inconsistent results can be seen for manure application as well (Afreh et al., 2018). Therefore, in addition to evaluating soil fertility and crop yields, environmental impacts need to be taken into account when assessing the performance of different organic materials returned to the field as well. It is necessary to comprehensively analyze the effects of organic matter returned to the field on crops, whether it improves nitrogen uptake by crops, and whether it contributes to the emission of N_2_O. Currently, there is a deficiency in research that evaluates different types of organic matter returned to the field in a holistic manner.

The North China Plain (NCP) is one of the dominant crop-producing regions in China, accounting for 71% and 55% of the country’s total production of wheat and maize, respectively (Shao et al., 2016). Nevertheless, high N application did not bring the expected high nitrogen use efficiency, and N recovery for the wheat–maize system in the NCP was only 16%–18% on average (Cui et al., 2010). Therefore, applying organic materials returning to the field to partly replace chemical fertilizer, especially for nitrogen fertilizer is necessary for the NCP. Meanwhile, a comprehensive assessment of its effects on crop yield, nitrogen use efficiency, and N_2_O emissions and evaluate potential trade-offs are needed. Hence, we assume that the partial substitution of chemical nitrogen fertilizers with organic materials for returning to the field can effectively augment crop yields, enhance nitrogen use efficiency, and mitigate N_2_O emissions. The objectives of this study are to: (1) quantitatively analyze the effect of different organic materials on the yield, soil nitrogen content, and the nitrogen recycling rate by plants for the wheat–maize cropping system; (2) discuss the effect of different organic materials on N_2_O emissions; and (3) select the optimal organic material to partly replace inorganic nitrogen fertilizer in the NCP. The findings of this study can further provide a basis for N fertilizer optimization measures in the NCP.

## 2 Materials and methods

### 2.1 Study site description

**Figure 1:**
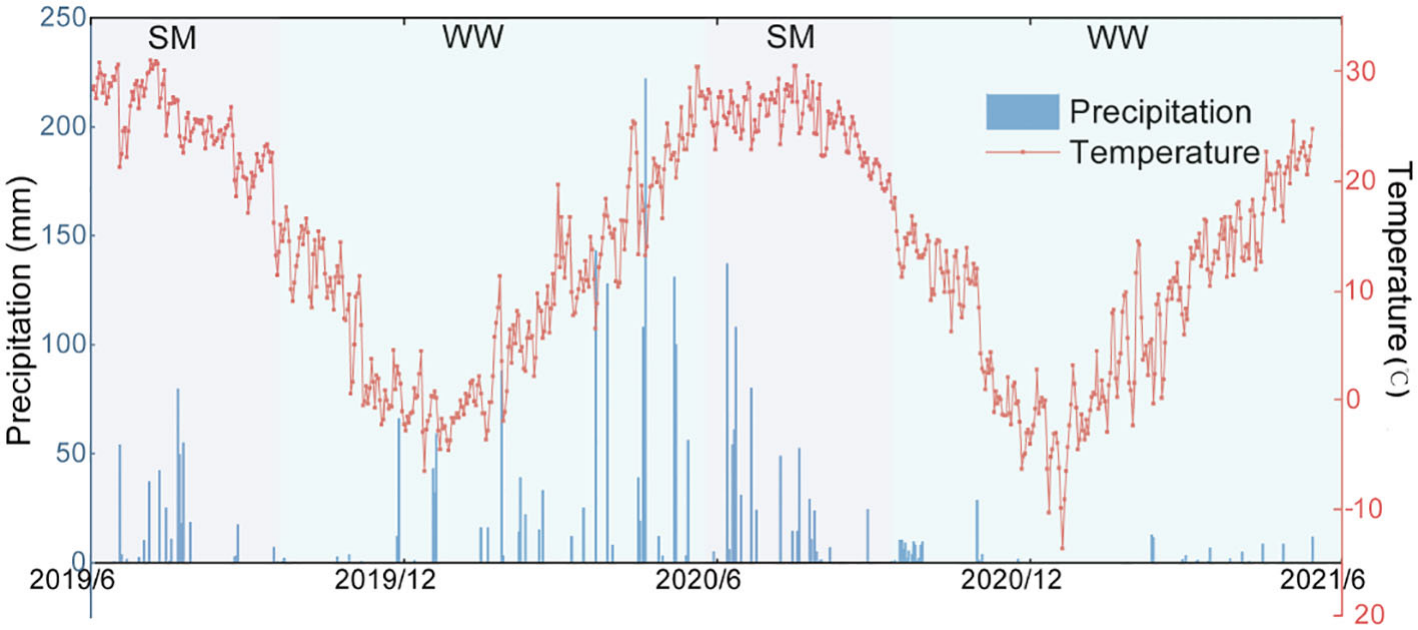
Daily mean temperature (dots) and monthly cumulative precipitation (bars) from June 2019 to June 2021 for the study site. SM, summer maize; WW, winter wheat.

This trial was conducted from 2019 to 2021 in Qianli Village, Wuqiao County, Hebei Province, China (37°41′N, 116°37′E). The area has a warm-temperate monsoon climate with an annual cumulative temperature (≥ 0°C) of 4,898°C and an annual mean precipitation of 562 mm. The meteorological data during the experimental period are shown in **Figure 1**. The soil classification of the test site was alluvial salt tide soil, categorized as chalky loam. Before the test started, the basic physical and chemical properties of the soil were assessed with (1) 0–10 cm: capacity of 1.31 g cm^−3^, total nitrogen of 1.03 g kg^−1^, organic matter of 16.07 g kg^−1^, effective phosphorus of 8.28 mg kg^−1^, and effective potassium of 198.96 mg kg^−1^; (2) 10–20 cm: capacity of 1.25 g cm^−3^, total nitrogen of 0.89 g kg^−1^, organic matter of 12.99 g kg^−1^, effective phosphorus of 7.32 mg kg^−1^, and effective potassium of 151.94 mg kg^−1^.

### 2.2 Experimental design and farmland management

**Figure 2:**
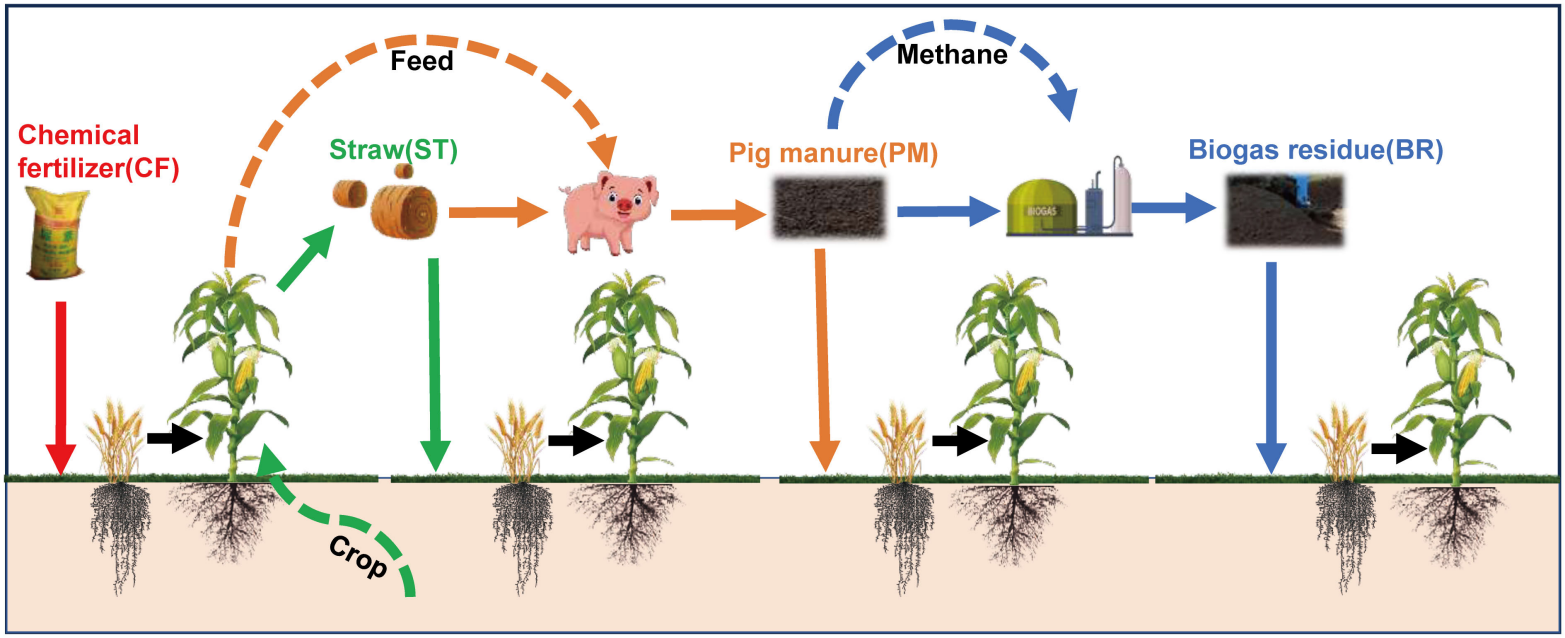
Experimental design concept diagram. The lines represent different segments of the circular agriculture system and the treatment of field return. The red line represents the chemical fertilizer treatment applied alone; the green line represents the farming production and straw treatment; the orange line represents the pig farming link and pig manure treatment; and the blue line represents the biogas fermentation link and biogas residue treatment.

The thinking of circular agriculture was applied in the experimental design. In the circular agriculture model, the straw residual can be used as pig feed, the pig manure can be used to produce biogas, and all the organic waste can be returned to the field. Therefore, winter wheat-summer maize with three organic materials added, straw (ST), pig manure (PM), and biogas residue (BR), as partial replacements of inorganic fertilizer, with chemical fertilizer only (CF) and no N application (WN) as control, were set in this experiment. The experimental design is shown in **Figure 2**.

The experiment follows the principle of equal nitrogen content, and a total of 150 kg N ha^−1^ was applied for the wheat and maize seasons each. For ST treatment, the solid biomass of straw from the previous season’s crop was returned to the field, where the N content of straw was calculated, and the additional N was replenished with chemical fertilizers. Prior to wheat sowing, the previous season’s maize straw was selected for incorporation into the field, with the incorporation rate calculated based on biomass and straw nitrogen content, resulting in a corn straw incorporation rate of 3,014 kg hm^−2^ (dry weight). Similarly, before maize sowing, the previous season’s wheat straw was selected for incorporation, yielding a wheat straw incorporation rate of 7,218 kg hm^−2^ (dry weight). For PM and BR treatment, the N application was 3:1 from organic materials to chemical fertilizer. The application rates of pig manure and biogas residue to the field were 1,281 kg hm^−2^ and 16,741 kg hm^−2^, respectively (dry weight). Organic materials, chemical phosphate fertilizer, potassium fertilizer, and half N fertilizer were applied as base fertilizer. The other half of the chemical N fertilizer was applied at the jointing stages. The detailed fertilization program is summarized in **Table 1**, and the properties of the organic material added are illustrated in **Table 2**.

**Table 1:** Fertilizer application scheme for different treatments.

Treatment	N (kg ha^−1^)				P_2_O_5_ (kg ha^−1^)	K_2_O (kg ha^−1^)
	Organic materials N	Base N	Top dressing N	Total		
ST	28	103.25	18.75	150	26	124
PM	112.5	18.75	18.75	150	26	124
BR	112.5	18.75	18.75	150	26	124
CF	0	75	75	150	26	124
WN	0	0	0	0	26	124

Nitrogen fertilizer uses urea containing 46.4% pure nitrogen, phosphate fertilizer uses diammonium phosphate containing 46% pure phosphorus and 18% pure nitrogen, and potassium fertilizer uses potassium sulfate containing 50% pure potassium. WN treatment phosphorus fertilizer is calcium superphosphate. ST, straw; PM, pig manure; BR, biogas residue; CF, chemical N fertilizer; WN, without N application.

**Table 2:** Properties of different organic materials.

Organic materials	C%	N%	Hemicelluloses%	Cellulose%	Lignin%	C/N	Lignin/N
Wheat straw	37.00	0.38	30.27	36.69	7.58	97.37	19.96
Maize straw	39.28	0.91	25.55	34.13	4.20	43.27	4.63
Pig manure	25.00	2.51	20.48	6.38	5.14	9.96	2.05
Biogas residue	26.00	1.92	14.47	6.78	1.00	13.54	3.47

ST, straw; PM, pig manure; BR, biogas residue; CF, chemical N fertilizer; WN, without N application.

The experiments were conducted in a randomized block design, with each treatment having three replications and a plot area of 5 m × 7 m. A variety of Jimai 22 was utilized for winter wheat at a sowing rate of 300 kg ha^−1^. Variety of Zhengdan 958 for summer maize was seeded with a row spacing of 50 cm and seed spacing of 30 cm. Irrigation and fertilizer application rates were generally consistent over the years. Wheat and maize were irrigated before planting and during elongation with 75 mm of water each time, respectively.

### 2.3 Plant sampling and plant N uptake calculation

During physiological maturity, a representative 1 m^2^ for wheat and a 5-m double row for maize were selected from each plot to calculate the grain yields randomly, with marginal rows avoided for the consideration of edge effect. After natural crop drying, the grain samples were weighed separately and calibrated by moisture content to determine the seed yield. Three maize plants and 1 m double-row wheat were randomly selected from each plot to determine the N content of plants and grains after air-drying and crushed through a 0.25-mm sieve. The N recovery efficiency (RE_N_, %) was calculated as follows:


(1) $${\text{RE}}_{N}=\frac{N_{uptakeT}-N_{uptakeN0}}{N}\times 100\%$$

where $N_{uptakeT}$ and $N_{uptakeN0}$ denote N uptake for N application with and without N application treatments, respectively, and N denotes the amount of total N applied to both organic materials and chemical fertilizers.

The N partial factor productivity (NPFP, kg kg^−1^) was formulated as follows:


(2) $$NPFP=\frac{Yield}{N}$$

where NPFP indicates the yield to total N applied to organic materials and chemical fertilizers ratio for each N application treatment.

### 2.4 Soil sampling and measurement

Soil samples for a 0–20-cm layer were obtained from each plot during the wheat and maize harvesting periods in June and October, respectively, using the five-point sampling method. After removing impurities such as plant residues, the soil samples were divided into two parts: one part passed through a 2-mm sieve and stored in a refrigerator at 4°C for the determination of nitrate and ammonium nitrogen within a week; the other part air-dried and passed through a 0.25-mm sieve for the determination of other indicators. Soil and plant total nitrogen (TN) was determined using the Kjeldahl method. Soil nitrate-nitrogen (NO_3_^−^-N) and ammonium nitrogen (NH_4_^+^-N) were determined by a continuous flow analyzer (AA3, SEAL, Germany).

### 2.5 N_2_O gas sampling and index calculation

Soil N_2_O emissions were calculated using the static chamber method (Cui et al., 2019; Zhao et al., 2021b). An open rectangular base (polymethylmethacrylate, 5 cm high) was randomly placed in each plot with a 20-cm × 20-cm × 30-cm greenhouse gas collection chamber cover. A geothermometer was installed next to the gas measurement base to measure the soil temperature at a depth of 5 cm at the time of gas extraction. After attaching the chamber lid to the base, 35 ml of gas samples were collected with a 50-ml polypropylene injector at 0-, 8-, 16-, and 24-min intervals in the middle of the static chamber and then transferred to pre-evacuated 12 ml glass vacuum collection tubes. These tubes were sent to the laboratory to determine N_2_O concentrations using a gas chromatograph (Shimadzu GC-2014C, Japan). The frequency of gas collection was usually once a week and increased after fertilization, precipitation, and irrigation, with 1, 3, 6, and 9 days after fertilization and rainfall/irrigation. The N_2_O flux (*F*, mg m^−2^ h^−2^) was calculated as follows:


(3) $$F=\frac{M}{V_{0}}\;\frac{V}{A}\;\frac{dc}{dt}\;\frac{T_{0}}{T}$$

where *M* denotes the relative molecular mass, *V_0_* is the volume of an ideal gas, *V* is the volume of the static box, and *A* is the area of the base, $\frac{dc}{dt}$ is the slope change of the gas concentration in the static chamber, *T* and *T*_0_ are room temperature and ideal gas temperature, respectively.

The cumulative N_2_O emissions (*T*, kg ha^−1^) were formulated in the following equation:


(4) $$T=\sum \left[\frac{\left(F_{i+1}+F_{i}\right)}{2}\times \left(t_{i+1}-t_{i}\right)\right]\times 24\times 10^{5}/10^{6}$$

where *F_i + 1_* and *F_i_* represent the N_2_O flux of two adjacent samples, *T_i + 1_* and *T_i_* are two corresponding sampling dates, 24, 10^5^, and 10^6^ represent conversions from h^−1^ to day^−1^, m^−2^ to ha^−1^, and mg to kg, respectively.

To better compare the effects of different organic materials on N_2_O emission, the yield-scald N_2_O emissions (YSNE_S_, g kg^-1^) were introduced in this study and formulated as follows:


(5) $${\text{YSNE}}_{S}=\frac{N_{2}O_{accumulation}}{Yield}$$

where ${\text{YSNE}}_{S}$ is the ratio of N_2_O accumulation to yield.

### 2.6 Statistical analysis

SPSS software and R were applied to analyze the differences in data between treatments. A one-way ANOVA was performed on the data to compare variability among the five treatments. A repeated measures ANOVA was performed on the data to compare differences between treatments and crops, and other indexes: treatment and crop were used as fixed effects, and random factors were used for sampling dates. R, GraphPad Prism, and Origin were used for graphing.

## 3 Results

### 3.1 Crop yield

Crop yields heavily relied on fertilization, and returning organic materials partly released chemical fertilizer, significantly changing crop yields (**Table 3**). Compared to WN, nitrogen application significantly increased the mean annual crop yields by 6.66%, 15.95%, 6.34%, and 12.03% for ST, PM, BR, and CF, respectively (*p* < 0.05). The PM showed the best performance among all treatments for both years. The yield for PM is 12.10% and 9.96% higher, respectively, than that for ST and BR in 2019–2020, and the higher ratios are 5.31% and 8.07%, respectively, in 2020–2021. The CF was better than BR and ST for the first year but had no difference with BR and ST for the second year. The effects of organic materials amendment on crop yield differ for wheat and maize. For maize season, PM and CF significantly increased maze yield compared to WN, and there were no significant differences among ST, BR, and WN in 2019. Except for ST, PM, BR, and CF significantly increased maze yield compared to WN in 2020 as well. The differences were more significant in wheat season than in maize season. All fertilization treatments increased wheat yield compared to WN in both years with PM being the highest. Compared to CF, the PM significantly improved wheat yield in both yields. The wheat yield for ST was lower than CF in the 2019–2020 season and significantly higher than CF in the 2020–2021 season. As for BR treatment, the wheat yield was significantly lower than CF in the 2019–2020 season but no difference with CF in the 2020–2021 season.

**Table 3:** Crop yields of different treatments for the wheat–maize system.

Year	Treatment	Maize (kg ha^−1^)	Wheat (kg ha^−1^)	Total annual production (kg ha^−1^)
2019–2020	ST	7,333 b	6,863 c	14,196 b
	PM	8,073 a	7,841 a	15,914 a
	BR	7,503 b	6,969 c	14,473 b
	CF	8,497 a	7,464 b	15,961 a
	WN	7,153 b	6,594 d	13,748 c
2020–2021	ST	6,340 bc	7,840 b	14,180 b
	PM	6,853 a	8,079 a	14,932 a
	BR	6,567 ab	7,251 c	13,817 b
	CF	6,664 ab	7,178 c	13,842 b
	WN	6,009 c	6,846 d	12,855 c

ST, straw; PM, pig manure; BR, biogas residue; CF, chemical N fertilizer; WN, without N application. Different letters in the same column indicate significant differences (p < 0.05).

### 3.2 Soil nitrogen content

**Figure 3:**
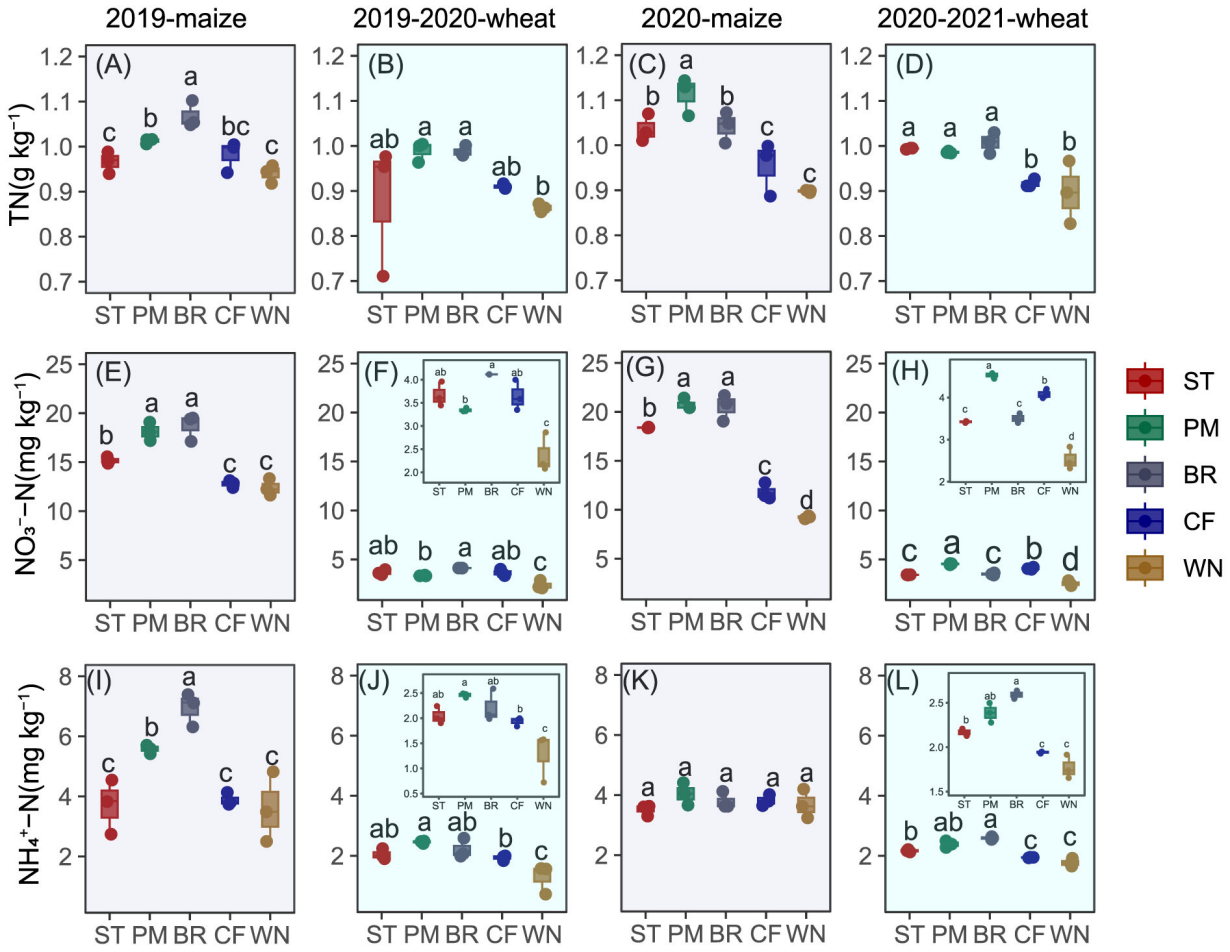
Soil total nitrogen **(A–D)**, nitrate nitrogen **(E–H)**, and ammonium nitrogen **(I–L)** contents in maize and wheat seasons from 2019 to 2021.

Soil nitrogen content was balanced by the nitrogen input and output. The application of exogenous nitrogen increases soil nitrogen content. Compared to WN, nitrogen application significantly increased the average soil total nitrogen content during four sampling periods by 7.75%, 13.97%, 14.09%, and 4.57% for ST, PM, BR, and CF, respectively (*p* < 0.05). Agricultural organic waste partial replacement of inorganic nitrogen fertilizer significantly increased soil nitrogen content (**Figure 3**), compared to inorganic nitrogen application alone. In comparison to CF, the soil TN content was not significantly changed in ST treatment, whereas in PM and BR treatments, significant increases of 8.99% and 9.10%, respectively, were observed (*p* < 0.05). Nitrate and ammonium N are available nutrients in soil that can be directly absorbed by plants. The influence of exogenous nitrogen input from chemical fertilizers and agricultural organic waste on soil inorganic nitrogen content was similar to its effect on TN. Compared to CF, the ST treatment increased soil NO_3_^−^ by 25.87% and had no significant effect on ammonium N. PM and BR treatments increased soil NO_3_^−^ by 44.67% and 44.93%, and soil NH_4_^+^ by 24.90% and 34.13%, respectively. Among the three materials, BR treatment was the most favorable for the increase of soil total, nitrate and ammonium nitrogen, followed by PM treatment.

Inorganic N content was significantly lower in wheat season than in maize season for all treatments. NO_3_^−^ content was 348.24% higher on average in maize season, and NH_4_^+^ was 104.66% higher than that in wheat season. The BR treatment was 414.45% higher in NO_3_^−^ content in maize season and 123.29% higher in NH_4_^+^ content in maize season than that in wheat season, and the BR treatment had the largest difference in inorganic N among all treatments in the maize and wheat seasons, followed by the PM treatment. Soil inorganic nitrogen content did not differ significantly between years.

### 3.3 N_2_O fluxes and accumulation

**Figure 4:**
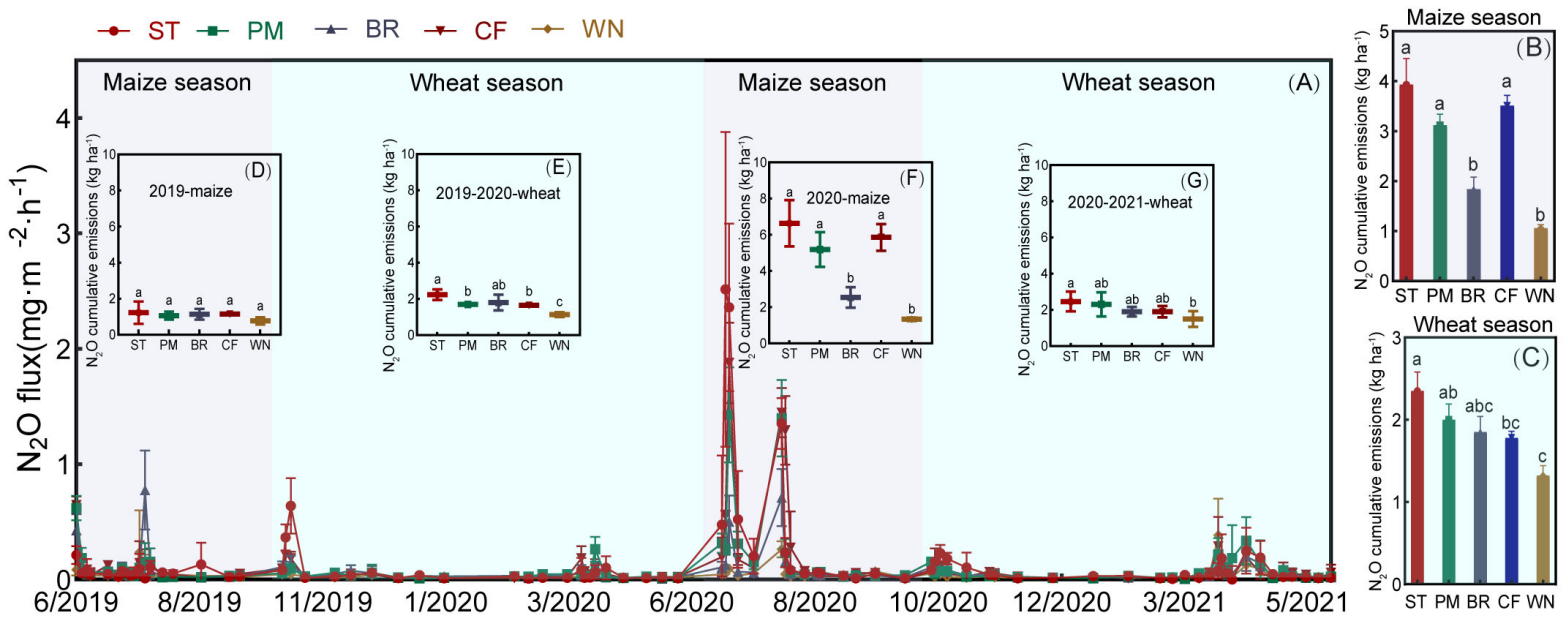
N_2_O fluxes from five treatments (ST, PM, BR, CF, WN) for the maize and wheat seasons for 2019–2021 **(A)**. Cumulative emissions for the maize season **(B)** and cumulative emissions for the wheat season **(C)** averaged over 2 years, and cumulative emissions for the 2019 maize season **(D)**, 2019–2020 wheat season **(E)**, 2020 maize season **(F)**, and 2020–2021 wheat season **(G)**.

The addition of exogenous nitrogen fertilizer stimulated soil N_2_O emission. Different organic materials application significantly altered N_2_O emission (**Figure 4**). The ST, PM, BR, and CF treatment significantly increased the cumulative N_2_O emission by 165.02%, 116.26%, 55.86%, and 122.82%, respectively, compared with WN (*p* < 0.05). For the treatment with three organic materials, ST treatment stimulated N_2_O emissions and BR treatment suppressed N_2_O emissions compared to CF. N_2_O emission fluxes ranged from 0.01 to 0.64 mg m^−2^ h^−1^, peaking after fertilization and irrigation. The emission peaks basically showed a trend of ST > CF > PM > BR, except for the emission peak after fertilization in wheat season, which showed a trend of PM > CF > ST > BR. Cumulative N_2_O emissions in 2019–2020 were significantly lower than in 2020–2021, and cumulative N_2_O emissions for ST, PM, BR, and CF treatments in 2020–2021 increased by 162.87%, 171.78%, 51.07%, 177.32%, and 48.29%, respectively, compared to 2019–2020.

Average N_2_O fluxes were significantly higher in the maize season than that in the wheat season. Cumulative N_2_O emissions during the maize season ranged from 1.06 to 3.93 kg ha^−1^, with the highest cumulative emissions from the ST treatment (3.93 kg ha^−1^), lower cumulative emissions from the PM and BR treatments than from the CF treatment (3.51 kg ha^−1^), and the lowest cumulative emissions from the WN treatment (1.06 kg ha^−1^). Cumulative emissions during the wheat season ranged from 1.32 to 2.35 kg hm^−1^, with ST (2.35 kg ha^−1^) treatment having the highest cumulative emissions, but PM (2.00 kg ha^−1^) and BR (1.85 kg ha^−1^) being higher than CF treatment (1.78 kg ha^−1^).

### 3.4 Interaction between grain yield and N utilization and emission

**Figure 5:**
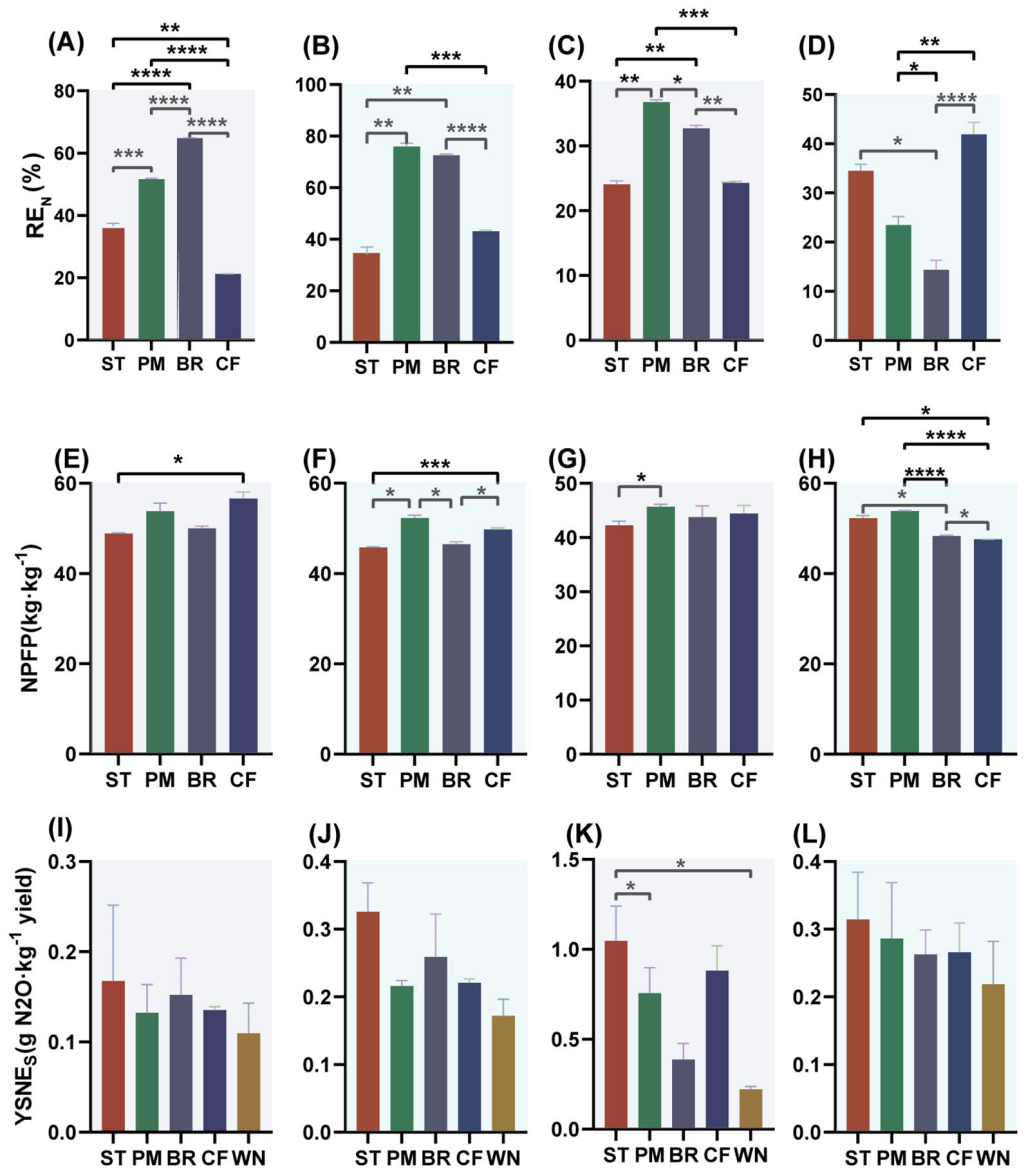
N recovery efficiency **(A–D)**, nitrogen partial factor productivity **(E–H)**, and yield-scaled N_2_O emissions **(I–L)** for the 2019 maize season, 2019–2020 wheat season, 2020 maize season, and 2020–2021 wheat season, respectively. Note: T, treatment; C, crop; D, sample date. Asterisks denote significance: ^*^ *p* < 0.05; ^**^ *p* < 0.01; ^***^ *p* < 0.001; ^****^ *p* < 0.0001.

Different organic materials returned to the field significantly affected the utilization of nitrogen fertilizer (**Figure 5**). RE_N_ is the proportion of nitrogen fertilizer applied in the farmland that is absorbed and utilized by the crop. The higher RE_N_ represents the sufficient utilization of the fertilizer application effect by the crop. NPFP is the ratio of the yield to the amount of nitrogen applied per unit area of the farmland. The key to improving NPFP lies in rational fertilization. YSNE_S_ is the ratio of N_2_O emission to yield, and its higher value represents higher N_2_O emission per unit of yield. Different organic materials have different effects on nitrogen fertilizer utilization and YSNE_S_. PM performed best among the three materials in terms of nitrogen utilization and reduced YSNE_S_. Compared to CF, ST significantly increased 23.25% YSNE_S_; PM significantly increased RE_N_ (44.01%) and NPFP (3.63%) and significantly decreased 7.49% YSNE_S_; BR significantly increased 41.39% RE_N_ and significantly decreased 4.96% NPFP and 29.40% YSNE_S_ (*p* < 0.05).

The effects of different organic fertilizers returned on nitrogen utilization in maize and wheat varied across seasons, while RE_N_ also varied between years, with RE_N_ significantly higher in the 2019–2020 than in the 2020–2021; RE_N_ trended lower in the maize season than in the wheat season. Compared to CF, ST significantly increased RE_N_ (71.95%), significantly decreased NPFP (13.69% and 8.06%) in the 2019 maize season, significantly increased NPFP (9.78%) in the 2020–2021 wheat season, and significantly increased YSNE_S_ (18.60%) in the 2020 maize season; PM significantly increased RE_N_ (142.93%, 76.61%, and 51.42%) in the 2019 maize season, 2019–2020 wheat season, and 2020 maize season and NPFP (13.12%) in the 2020–2021 wheat season, which was not significantly different from YSNE_S_ in CF treatment; BR treatment significantly increased RE_N_ (205.02%, 68.71%, and 34.61%), significantly decreased RE_N_ (65.73%) in the 2020–2021 wheat season, and significantly decreased NPFP (6.63%) in the 2019–2020 wheat season and decreased NPFP (1.53%) in the 2020–2021 wheat season, which were not significantly different from CF treatment YSNE_S_ (*p* < 0.05).

**Figure 6:**
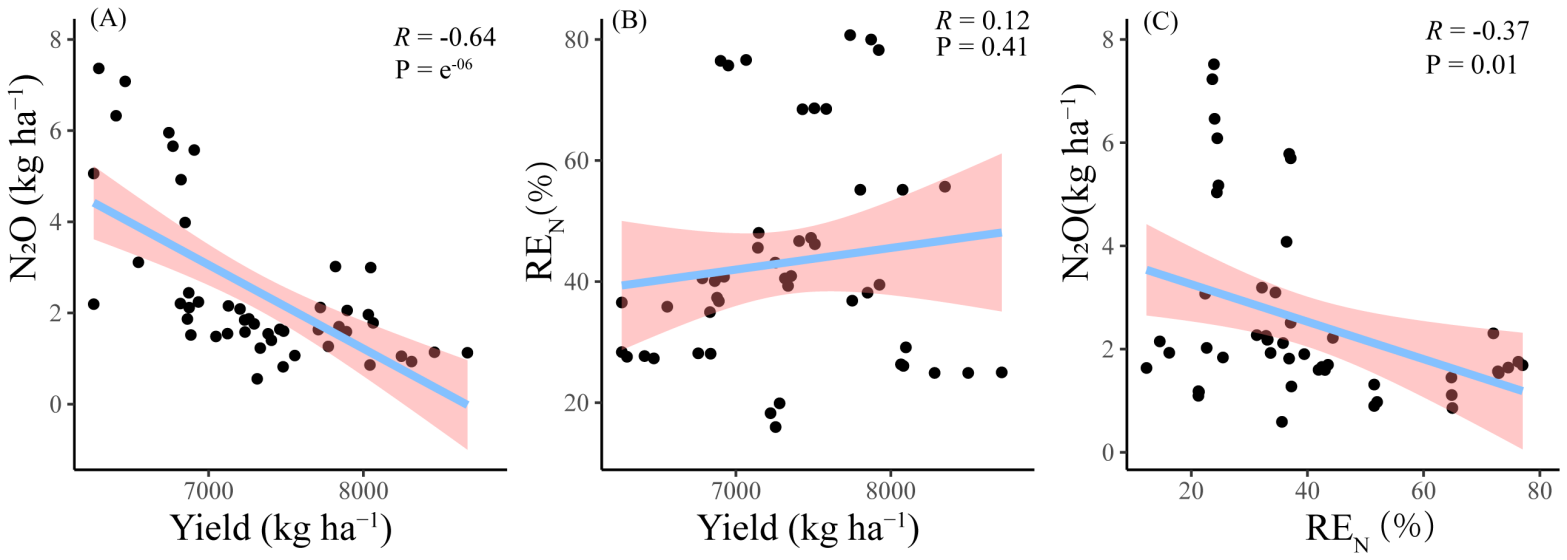
Correlations between N_2_O and yield **(A)**, RE_N_ and yield **(B)**, and N_2_O and RE_N_ **(C)**. “−” indicates negative correlations.

Crop yield, N recovery efficiency, and N_2_O emission interacted with each other (**Figure 6**). Pearson correlation analysis showed that the increase in yield was accompanied by the decrease in N_2_O emission, and there was a highly significant negative correlation between yield and N_2_O emission (*R* = − 0.64), and there was a significant negative correlation between RE_N_ and N_2_O emission (*R* = − 0.37), with the higher the RE_N_, the lower the N_2_O emission. Meanwhile, the increase of RE_N_ might be accompanied by the increase of yield, but the relationship was not significant.

### 3.5 Synchronization of yield increase, N utilization improvement, and N_2_O emission mitigation

**Figure 7:**
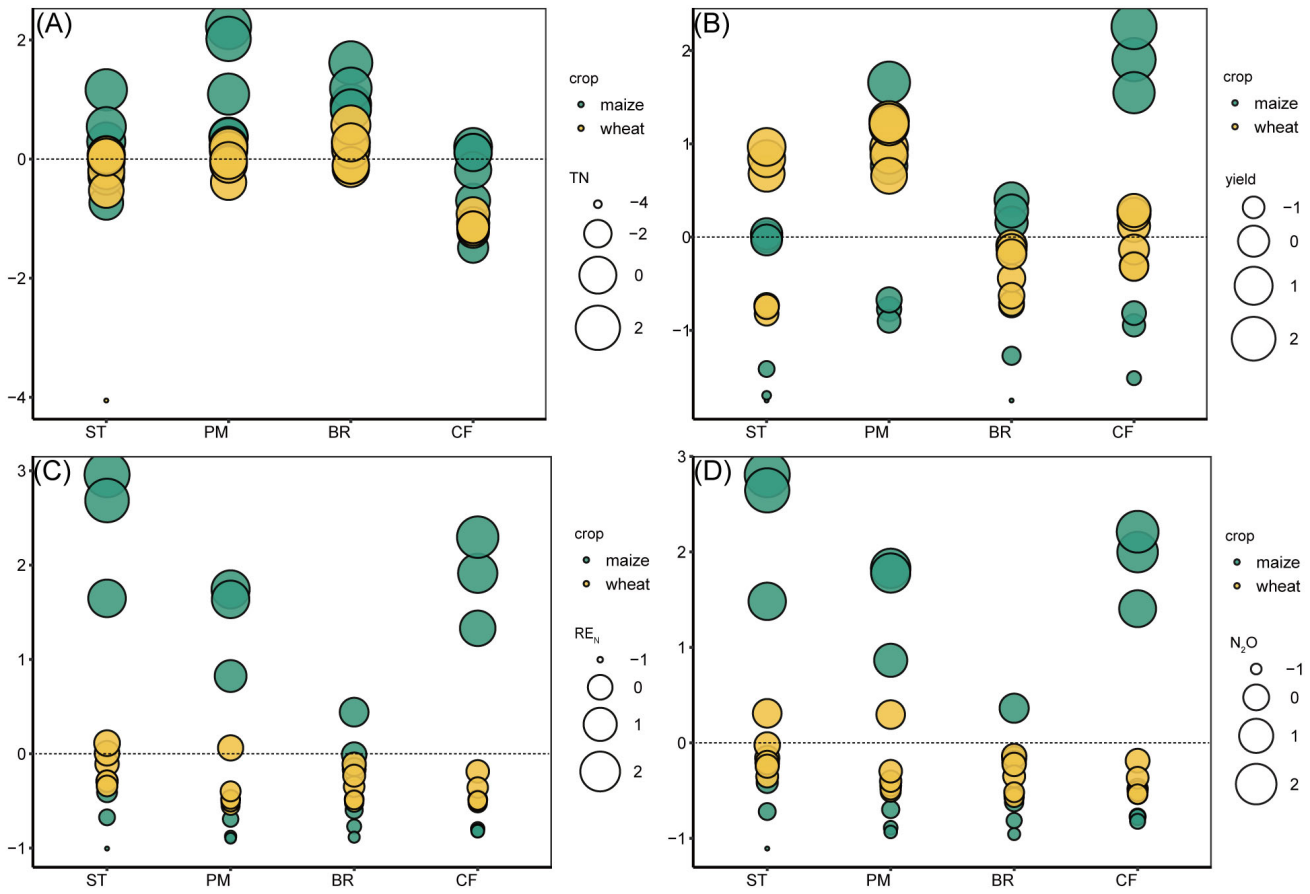
*Z*-Score results of TN **(A)**, yield **(B)**, RE_N_ **(C)**, and N_2_O **(D)** under ST, PM, BR, and CF treatments.

Considering the *Z*-score for TN, yield, RE_N_, and N_2_O emissions after organic materials returned, different organic materials applied affected the combined N utilization, and the effects of different organic materials on nitrogen synthesis were inconsistent across crops (**Figure 7**). Soil total nitrogen content, RE_N_, and yield were significantly superior to those of applied inorganic fertilizers alone after organic materials were returned to the field, and N_2_O emission was not altered by the other two types of organic materials returned to the field, except for straw, which stimulated N_2_O emission. Among the three organic materials, PM treatment had the best effect on TN accumulation, yield increase, and RE_N_ increase, followed by BR treatment, and ST had the worst effect. Soil TN and N_2_O emissions were significantly higher in the maize season than in the wheat season, and the performance of different organic materials was inconsistent between the maize and wheat seasons. The overall most effective pig manure returned to the field increased soil TN in the wheat and maize seasons, increased wheat yield, RE_N_ in the maize and wheat seasons, and suppressed N_2_O emissions in the maize season. The least effective straw treatment for the field returned reduced maize yield, stimulated N_2_O emissions in the maize and wheat seasons, and reduced wheat season RE_N_.

## 4 Discussion

### 4.1 Effect of organic materials on yield and N utilization

In terms of yield, we can clearly see the strong dependence of crop yield on nitrogen and the significant effect of different organic materials returned to the field on yield (**Table 3**). The results of our study showed that straw returns and biogas residue returns reduced crop yields, and pig manure returns increased crop yields. Earlier research about the influence of straw return on crop yield showed different results. Previous studies have shown that crop residue return is an effective technique for increasing crop yields and improving soil properties and plays a critical role in sustainable agricultural systems (Liu et al., 2019a; Zhao et al., 2020b). However, in contrast to conventional straw return measures, our study was conducted based on replacing inorganic nitrogen fertilizer with nitrogen from returned straw. Straw recycling may stimulate the crop mineral nutrients, but the short-term decomposition of crop straw inputs may lead to microbial N assimilation and limit the effectiveness of soil N (Shen et al., 2014). As for the return of pig manure and biogas residue to the field, the results of a meta-analysis showed that the return of pig manure to the field increased grain yield by 7.68%, while the return of biogas residue to the field decreased the grain yield (Fan et al., 2021). Our study yielded similar results. The possible reasons for the increase in wheat and maize yield by pig manure are as follows: Firstly, besides mineral N, other mineral nutrients such as phosphorus, potassium, calcium, magnesium, and sulfur are added through the application of animal manure (Gil et al., 2008; Singh et al., 2019). Secondly, the application of pig manure enhanced the availability of soil moisture and nutrients, thereby increasing the absorption ability of the root system (Su et al., 2006; Liu et al., 2010). Third, with the extra application of organic fertilizers, the development and multiplication of soil microorganisms were stimulated, soil microbial communities were improved, and the diversity of bacteria was enriched, thus raising the productivity of the soil (Liang et al., 2009). Whereas the biogas residue has a lower content of organic carbon than the other types of organic material, which influences the fertility of the soil (Fan et al., 2021). Meanwhile, our results also showed that the yield of organic materials continued to increase after returning to the field as the years of returning to the field increased, and in the short term, the yield depended on the input of chemical nitrogen fertilizers because of the slow decomposition rate of organic materials and the slow release of nutrients (Ge et al., 2021), but in the long term after returning to the field, the yield continued to increase with the decomposition of organic materials and the accumulation of soil nutrients, and Shen et al. (2014) showed that the negative effects of straw on crop productivity could be compensated by the continuation of straw return. Also, pig manure can provide effective N in the initial phase of field return because of its higher rate of nutrient release and N conversion than straw and biogas residue. At the same time, straw and biogas residue reduced maize yields, and organic materials had a better effect on wheat yields, also because of material decomposition and nutrient release, since wheat has a long reproductive period and organic materials such as straw do not decompose completely during the maize growing period.

Returning organic materials to the field significantly enhanced soil nitrogen levels and increased nitrogen fertilizer recycling efficiency (**Figures 3**, **5**). Among the three organic materials, biogas residue effectively increased soil TN, and pig manure significantly increased NPFP and RE_N_. Xie et al. (2022) also found that manure application was an eco-friendly strategy to improve yield, N uptake and N use efficiency, and soil fertility. The results of the study by Marchezan et al. (2020) also showed that combining organic fertilizer (before sowing) application with mineral nitrogen fertilizer (topdressing) could maintain N effectiveness in the soil solution during the organic N demand period, promote N utilization, improve crop growth, and increase yield. It was shown in the study that straw played a negative role in RE_N_. Although the positive effects of straw return to the field on soil fertility and productivity have been widely reported, numerous studies have indicated that straw return has little or no negative effects on soil fertility and productivity. At the initial stage of field return, adding straw could fix the effective soil N, thus limiting crop production and N uptake. The phenomenon depended on the amount of straw incorporation (Malhi et al., 2011). In addition, soil nitrogen content and nitrogen fertilizer recycling efficiency differed between crops and years. Soil total, ammonium, and nitrate N were significantly higher in the maize season than in the wheat season, N fertilizer recovery efficiency was lower than in the wheat season, and N fertilizer partial productivity was higher than in the wheat season. RE_N_ and NPFP were higher in 2019–2020 than in 2020–2021 (**Figures 3**, **5**). During the maize growing season, the elevated precipitation and temperature levels, as compared to the wheat season, led to an enhancement in the rate of organic matter mineralization, which positively correlated with increasing temperature and humidity. Irrespective of the fertilizer application method employed, this environmental shift resulted in elevated concentrations of soil nitrate and ammonium nitrogen (Dromantiene et al., 2020). High precipitation in the maize season of 2020–2021 and high precipitation in the preceding period resulted in lower yields and biomass than those in 2019–2020, and consequently, lower RE_N_ and NPFP than those in 2019–2020. It is important to note that RE_N_ was significantly higher in the chemical nitrogen fertilizer alone treatment than in the organic materials return treatment in the 2020–2021 wheat season. The formation of RE_N_ is directly influenced by biomass and nitrogen content (Equation 1). The inconsistent trend of RE_N_ during the 2020–2021 wheat growing season compared to other crop growth periods primarily stems from variations in biomass. Soil fertility and moisture status are crucial for wheat growth and development. Under moderate soil fertility and adequate water supply, wheat can grow normally and accumulate sufficient biomass (Agegnehu et al., 2014). Pre-irrigation using sole chemical fertilizers enhances soil moisture to meet wheat’s growth demands and facilitates rapid biomass formation post-jointing stage irrigation and topdressing. Elevated temperatures during this period stimulate root growth and nutrient uptake in winter wheat, augmenting aboveground biomass (Drebenstedt et al., 2023). However, insufficient rainfall in later stages (**Figure 1**) disrupts material allocation and transformation mechanisms, adversely affecting yield formation. However, organic material treatments during wheat jointing apply lower topdressing amounts compared to sole chemical nitrogen fertilizer treatments, resulting in lower biomass accumulation. In cases of inadequate rainfall during the late jointing stage, organic material incorporation into the soil can optimize material allocation and transformation mechanisms, enabling higher yields despite relatively lower biomass. Consequently, under conditions where nitrogen content in wheat aboveground parts and other growth periods remain relatively unchanged, variations in precipitation and topdressing lead to lower biomass in organic material treatments compared to sole chemical nitrogen fertilizer treatments, ultimately contributing to differences in RE_N_.

### 4.2 Effect of organic materials on N_2_O emissions

Nitrogen fertilizer application significantly stimulated N_2_O emissions. Whether organic fertilizers increase N_2_O emissions compared to chemical fertilizers has been debated (Cayuela et al., 2010; Deng et al., 2012; Chen et al., 2014b; Zhou et al., 2017). In our study, straw application significantly increased N_2_O emissions, while the PM treatment was not significantly different from CF, and BR inhibited N_2_O emissions compared to CF (**Figure 4**).

Guo et al. (2021) also showed that straw return to the field significantly increased N_2_O emissions. In a dryland system such as a wheat field, soil N_2_O production is mainly controlled by the action of soil microbial growth and development, which are mainly influenced by soil temperature and humidity. The uniform placement of crop straw on the soil surface increases soil moisture and facilitates N_2_O emissions (Hu et al., 2019; Li et al., 2019). The straw return may increase soil N_2_O emissions by increasing the effectiveness of soil C and N substrates (Rizhiya et al., 2007). The straw return provided more metabolic substrates for nitrification or denitrification (Hu et al., 2019). Straw return may increase microbial effective carbon, promote microbial oxygen demand to conditions conducive to denitrification, and spur N_2_O release (Senbayram et al., 2012). Previous studies also reported a significant increase in initial N_2_O emissions from straw returns compared to nonstraw returns (Hu et al., 2019). This may be due to straw incorporation’s different C/N and soil N effectiveness (Hu et al., 2019). N_2_O emissions from pig manure and biogas residue were lower in the maize season with high precipitation than in the CF treatment (**Figure 4**), and this significant reduction may be due to the following reasons: Firstly, the organic nitrogen contained in both materials was partially and rapidly converted to inorganic nitrogen, and this alteration resulted in a reduction in the amount of substrate directly available to nitrifying and denitrifying microorganisms, thus reducing N_2_O production and emissions (Cai et al., 2009). Secondly, the degradation of organic matter exhausts the oxygen in the soil and depresses nitrification, thus reducing N_2_O emissions during N conversion (Zhang et al., 2021). Previous studies (Zeng et al., 2021) have shown that one possible reason for the peak N losses before and after fertilizer application may be the relatively low crop use efficiency of N during early crop growth. Ammonium N was the major form of N loss in maize fields, accounting for 50.27%–62.32% of TN losses. Dryland crops such as maize prefer to take up nitrate N rather than ammonium N as the main source of N (Zeng et al., 2021). Nitrate-N acts as a substrate for N_2_O production and yield enhancement, resulting in a competitive relationship between yield, nitrogen use, and N_2_O emissions. It was also shown that N_2_O emissions were higher in the maize season than in the wheat season (**Figure 4**) because of the increased effectiveness of soil N substrate decomposition, and high temperature and humidity promoted N_2_O emissions more in the maize season than in the wheat season (Cheng et al., 2023). Straw treatment for maize season emissions spiked because high temperatures and humidity promoted straw decomposition, releasing carbon and nitrogen and increasing soil N substrate. In contrast, the surge in emissions in the inorganic fertilizer treatment during the maize season was due to the inputs of chemical N fertilizers that directly provided substrates for N transformation and stimulated N_2_O emissions, whereas the transformation process was slow during the wheat season with low temperatures.

### 4.3 Synchronization of production increases, N utilization improvements, and N_2_O emission mitigation

The experiment was based on equal nitrogen application, and the total nitrogen was the same for each treatment except for the without-nitrogen treatment, the source of the differences produced being the different forms of nitrogen inputs. Since some of the nitrogen in the field return test program was supplemented by inorganic nitrogen fertilizers, there was almost no retention of inorganic nitrogen after it was applied to the soil, and the differences in soil total nitrogen between treatments were mainly due to the differences in the N content and form of the different organic materials (Zhao et al., 2021a). Among the three types of agricultural wastes used for field return, straw, pig manure, and biogas residue, straw had the highest cellulose, C/N, and lignin/N contents, followed by biogas residue, and pig manure was the lowest (**Table 2**), and nitrogen in pig manure was the most readily converted and utilized. When compared to the application of pig manure as a nitrogen supplement in agricultural fields, the use of biogas residue and straw for nitrogen supplementation demonstrates a delayed or lagging effect.

Our study found that pig manure with inorganic N fertilizer was the optimal fertilization option to improve crop yield and N use efficiency and did not increase or even mitigate N_2_O emissions. Substituting straw for inorganic N fertilizer reduced crop yield and N use efficiency, stimulating N_2_O emissions. Much research today has focused on improving yields and nitrogen use efficiency, reducing pollution, and lowering climate costs (Chen et al., 2014a). Straw return to the field has been the most common agricultural waste material due to experimental field operations and material availability, but due to its decomposition time and material characteristics, straw cannot be used as an inorganic nitrogen fertilizer replacement material. Simultaneously, pig manure can serve as an effective material for increasing yields and enhancing nitrogen utilization levels, all without exacerbating or even slowing down N_2_O emissions. After the straw return, a small amount of N was mineralizable, and the remaining N may lead to a significant accumulation of organic carbon in the soil, which in essence may affect N_2_O emissions and increase N_2_O emissions due to the additional N content. In contrast to inorganic N fertilizers, manure provides essential plant nutrients other than N and may help promote crop growth and nutrient uptake (Karimi and Akinremi, 2018). In addition, total N input from manure is likely to be higher than urea because urea N rates are determined by the fast-acting N in manure. Yield response also affects the intensity of N_2_O emissions (Adelekun et al., 2021). Previous studies have shown that N_2_O emissions from soils occurred when the effectiveness of N in the soil exceeded crop requirements, especially when N was recently added (Kudeyarov, 2020). However, as the crop matures throughout the growing season, N uptake by the crop increases, thereby reducing N losses as emissions.

## 5 Conclusion

Partial replacement of fertilizers with organic wastes is considered an option to promote green agricultural development in China. This study applied three organic materials, straw, pig manure, and biogas residue, to analyze their effects on crop production, N utilization, and N_2_O emissions. The results of this study showed that organic materials return increased TN and RE_N_ compared with inorganic nitrogen fertilizer alone. However, the addition of organic materials generally resulted in a reduction of crop yield by 4.79% to 5.08%, with the notable exception of pig manure, which was found to increase crop yield by 3.50%. Different treatments had different effects on RE_N_ and N_2_O emissions, with ST treatment being the least effective. ST reduced wheat RE_N_ by 19.35% and stimulated soil N_2_O emission. BR reduced yield and decreased N_2_O emission. Pig manure reuse increased soil RE_N_ (44.01%) and yield (3.50%) without increasing N_2_O emissions. From a crop perspective, organic materials stimulated N_2_O emissions during the wheat season, while pig manure and biogas residue suppressed N_2_O emissions in the maize season. Simultaneously, crop yields continued to increase with the number of years of organic materials returned to the field. This study emphasizes that pig manure return to the field is an important way for effective N fertilization to improve N-use efficiency and yield without exacerbating the pollution caused by N_2_O emissions.

## Data Availability

The original contributions presented in the study are included in the article/supplementary material. Further inquiries can be directed to the corresponding authors.
